# Pharmacological properties of *Centella asiatica* hydrogel in accelerating wound healing in rabbits

**DOI:** 10.1186/s12906-019-2625-2

**Published:** 2019-08-14

**Authors:** Afnan Sh. Ahmed, Muhammad Taher, Uttam Kumar Mandal, Juliana Md Jaffri, Deny Susanti, Syed Mahmood, Zainul Amiruddin Zakaria

**Affiliations:** 1 Department of Pharmaceutical Technology, Kulliyyah of Pharmacy, Faculty of Pharmacy, International Islamic University Malaysia Bandar Indera Mahkota, Jalan Sultan Ahmad Shah, 25200 Kuantan, Pahang Malaysia; 2 Maharaja Ranjit Singh Punjab Technical University, Punjab, India; 3 Department of Chemistry, Kulliyyah of Science,International Islamic University Malaysia, Bandar Indera Mahkota, 25200 Kuantan, Pahang Malaysia; 4 Department of Pharmaceutical Engineering, Faculty of Engineering Technology, University Malaysia Pahang, Pahang, Malaysia; 5 Laboratory of Halal Science Research, Halal Products Research Institute, Universiti Putra Malaysia, 43400 UPM, Serdang, Selangor Malaysia; 6 Integrative Pharmacogenomics Institute (iPROMISE), Faculty of Pharmacy, Universiti Teknologi MARA, Puncak Alam Campus, Bandar Puncak Alam, Selangor Malaysia

**Keywords:** Asiaticoside, *Centella asiatica*, Apiaceae, Hydrogel film, Wound dressing, Wound healing, PVA/PEG

## Abstract

**Background:**

Various extracts of *Centella asiatica* (Apiaceae) and its active constituent, asiaticoside, have been reported to possess wound healing property when assessed using various in vivo and in vitro models. In an attempt to develop a formulation with accelerated wound healing effect, the present study was performed to examine in vivo efficacy of asiaticoside-rich hydrogel formulation in rabbits.

**Methods:**

Asiaticoside-rich fraction was prepared from *C. asiatica* aerial part and then incorporated into polyvinyl alcohol/polyethylene glycol (PVA/PEG) hydrogel. The hydrogel was subjected to wound healing investigation using the in vivo incision model.

**Results:**

The results obtained demonstrated that: i) the hydrogel formulation did not cause any signs of irritation on the rabbits’ skin and; ii) enhanced wound healing 15% faster than the commercial cream and > 40% faster than the untreated wounds. The skin healing process was seen in all wounds marked by formation of a thick epithelial layer, keratin, and moderate formation of granulation tissues, fibroblasts and collagen with no fibrinoid necrosis detected.

**Conclusion:**

The asiaticoside-rich hydrogel developed using the freeze-thaw method was effective in accelerating wound healing in rabbits.

## Introduction

It is currently estimated that approximately six million people globally suffer from chronic wounds. Wound healing process is a defined biological procedure that regenerate tissues, it involves complex cascade of events that are divided into three main unique, yet overlapping, phases include inflammation, proliferation and maturation [1–5]. Furthermore, the wound healing mechanism encompasses complex interactions between the various cell types, the components of the extracellular matrix and the cytokine mediators [2, 4, 5]. Even though the natural wound healing mechanism to recover damaged tissues is initiated when a wound is introduced, yet a suitable dressing for the wounds should be used. To enhance the healing process, the dressing is ought to be able to intervene at the precise phase of wound healing or providing the right environment for the wound to heal [1, 6]. In general, a good and effective wound dressing ought to have the ability to maintain a moist wound environment, protect the wound from secondary infection, heal the wound faster, reduce wound bed necrosis and will not introduce a secondary trauma to the regenerated tissues once the dressing is removed from the healed wounds. Additionally the wound dressing should be biocompatible with the tissues as well as blood, not antigenic, not toxic and with suitable elasticity [1, 4, 6, 7]. In light with these requirements, biocompatible polymeric hydrogels can be the ideal promising wound dressing materials, as they fit with the effectual wound dressing requirements by providing an easy to handle dressing with no irritation and no adhering properties, hence maintain or enhance patients’ comfort [1, 6, 8]. In a previous study by the current authors, PVA/PEG hydrogel films were developed using the freeze-thaw technique [9]. Traditional herbal drugs have become the focus of the scientific researchers due to the fact that traditional medicines can offer a safe and inexpensive method for treating wounds and burns [10–13]. *Centella asiatica* extract showed its activity in tissue regeneration [14], cell migration [15] and wound repair process by promoting fibroblast proliferation and collagen synthesis [16]. The wound healing potential of *C. asiatica* extracts, or *‘pegaga nyonya’* as it is known to the Malay, have been justified in both experimental and clinical evaluations [17–21], with most of these studies reporting that asiaticoside as the main active constituent producing the said effect. *C. asiatica* also posseses several interesting phytochemical constituents such as flavonoids, sesquiterpenes, plant sterols, pentacyclic triterpenoids, eugenol derivatives and caffeoylquinic acids [22].

The wound healing activity of asiaticoside has been reported using in vivo and in vitro models [23–27]. The study was aimed to develop an asiaticoside-rich PVA/PEG hydrogel drug delivery formulation using the freeze-thaw technique with enhanced wound healing capability.

## Methods

### Materials

All the required chemicals and reagents used in the study were of analytical reagent grade. Asiaticoside standard and poly(vinyl alcohol) (PVA) (Mw 195,000) (Mowiol® 56–98) were purchased from Sigma-Aldrich (USA), and Polyethylene glycol 400 (PEG 400) from Merck (Germany). Silica gel 60 (70–230 mesh), methanol and hexane were purchased from Merck (Germany), dichloromethane (DCM) and ethyl acetate from Fisher Scientific (UK), ethanol from R&M Chemicals (UK).

For the animal studies, MADECASSOL® cream was purchased from Bayar (Turkey), Ketamine hydrochloride (100 mg/mL) and xylazine hydrochloride (100 mg/mL) were both purchased from Ilium (Australia) and normal saline-solution was bought from Opticare (Malaysia).

### Plant collection, extraction and fractionation

The plant (*C. asiatica*) was purchased from Lorong Kenanga, Kampung Pandan Dua, Kuantan, Pahang, Malaysia, on 19 April 2014. The plant was identified by Dr. Shamsul Khamis (taxonomist) from the Institute of Bioscience, University Putra Malaysia, Selangor, Malaysia. The plant was given a voucher specimen number (PIIUM 0205) and has been deposited at the Herbarium, Kulliyyah of Pharmacy, IIUM Kuantan, Malaysia, for future reference.

**Fig. 1:**
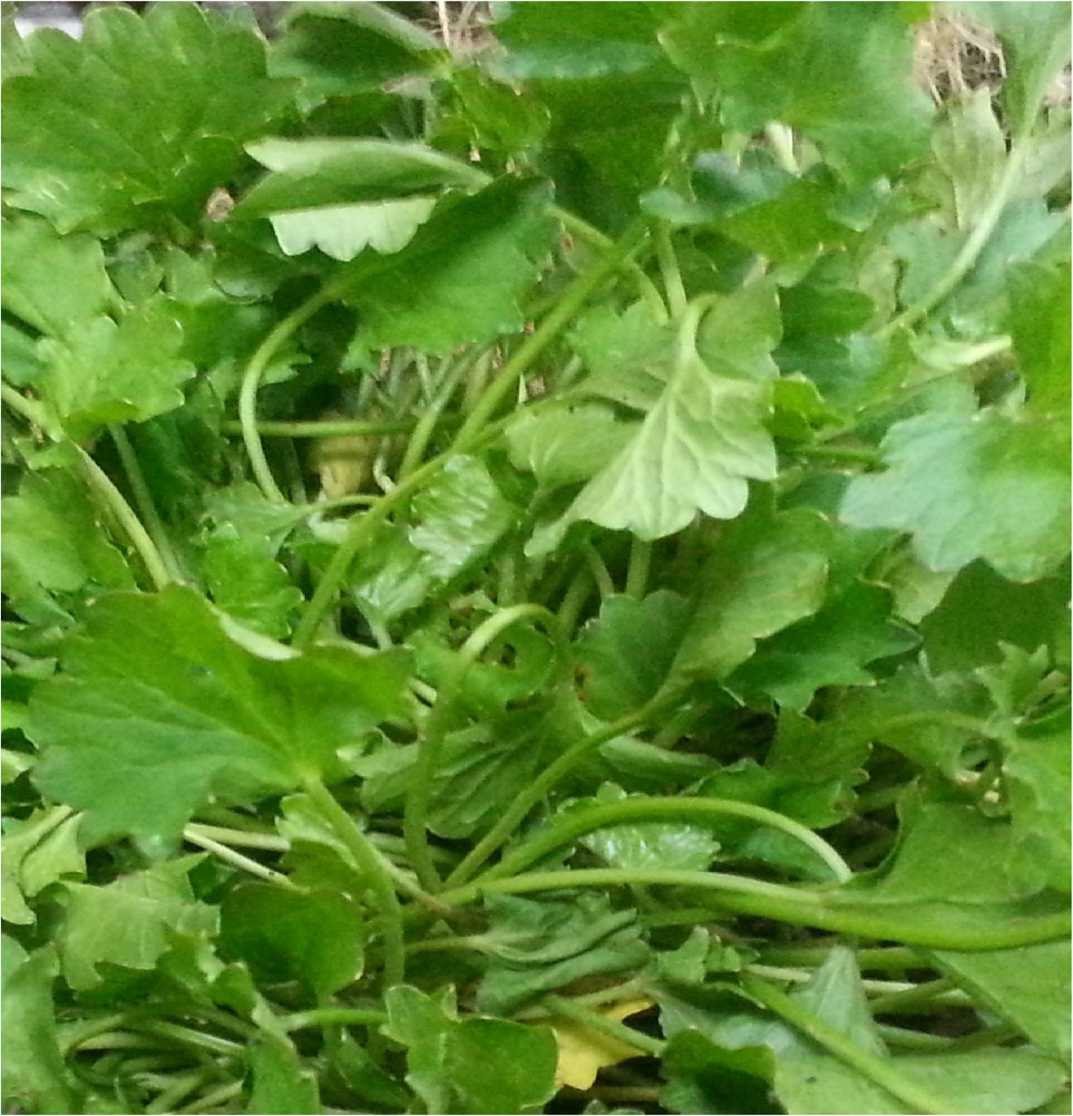
*Centella asiatica*

The extraction and preparation of asiaticoside-rich fraction was carried out according to the method described by Aziz et al. [15]. The aerial part of *C. asiatica* (Fig. 1) was collected and dried at a temperature of 35 to 40 °C for three days. The dried plant was pulverised. Approximately 100 g of the pulverised *C. asiatica* was macerated in 300 mL of 95% ethanol (1:3 w/v ratio) for a period of three days. Maceration was repeated thrice. The extract was obtained after filtering through Whatman No1 filter paper and was concentrated by Büchi rotary evaporator. Then the extracts were subjected to Vacuum Liquid Chromatographic (VLC) procedure. The solvent phase used consisted of 1. 100% hexane; 2. hexane- dichloromethane (DCM) (50:50); 3. 100% DCM; 4. DCM-ethyl acetate (50:50); 5. ethyl acetate; 6. 100% ethyl acetate-methanol (50,50) and 7. 100% methanol (CAEMF-asiaticoside-rich fraction). Thin layer chromatographic analyses of the fractions were carried out.

### Hydrogel formulation

The *C. asiatica* PVA/PEG hydrogel was prepared by the freeze-thaw method by Ahmed et al., [9]. First, PVA (8%) was dissolved in deionized water for 1 h on a hotplate and stirred using a magnetic stirrer. Then, PEG (5%) was added and the solution was autoclaved at 121 °C for 15 min. Next, the asiaticoside-rich fraction (fraction CAEMF/ (from fraction **7**) (24 mg of fraction) was dissolved in the above formulated PVA/PEG hydrogel and underwent five consecutive freeze-thaw cycles.

### Animal care

The animal study on rabbits began after ethics approval was obtained from the Institutional Animal Care and Use Committee, International Islamic University Malaysia (IACUC, IIIUM) (Ethics approval reference number: IIUM/IACUC Approval/ 2016/(9)(54)). New Zealand White Albino rabbits (6 males) with 1.8–2.1 kg body weight were purchased from a private company (Chenur Supplier Pvt. Ltd.) in Kuantan, Pahang, Malaysia. The rabbits used in this study were handled in accordance with accepted standards [28]. The rabbits were kept in the animal house division at Level 5, Kulliyyah of Pharmacy, IIUM for an acclimatization period of around 2 weeks. Each animal was placed in an individual aluminium cage during the study. The rabbits were kept in a holding room at room temperature of 19 ± 4 °C, 44 to 55% relative humidity and 12 h light/dark. The rabbits were fed with commercial pellets and fresh vegetable diet and also had access to water.

### Acute dermal irritation

Acute dermal irritation testing of the hydrogel samples was carried out according to OECD Guideline 404 [29].

### In vivo wound healing activity (incision wounds)

The wounds were divided into four groups in six rabbits as follows: Group 1: treated with asiaticoside-rich hydrogel, Group 2: treated with Madecassol cream (Bayer) (positive control), Group 3: treated with blank hydrogel, Group 4: no treatment (negative control). The treatment with the above dressings (Group 1 to 4) on the wounds were placed randomly on the back of the rabbit to avoid bias in the position of wounds. Before starting the incisional wound procedure, the animal hairs on the dorsal thoracic region were shaved and 70% ethanol was used as antiseptic for the shaved region [28]. Then, the incisional operation began by generally anaesthetizing the rabbits through a subcutaneous injection of Ketamine: Xylazine (0.4: 0.1 mL, 40: 10 mg/kg). A 1 cm long incision was made using a sharp scalpel [30]. The first day was counted as the wounding day (day 0). The wounds were treated once daily until the completed experimental day. At the end of the experiment (Day 12), all the *rabbits* were *euthanized* by a *lethal dose* of *pentobarbital* (200 mg/kg).

### Statistical analysis

The data were analysed using SPSS (IBM® SPSS® Statistics version 20, SPSS Inc., Chicago, IL, USA). One-way analysis of variance (One-way ANOVA) was used to compare the results and a *p* value of less than 0.05 was considered as statistically significant. The experiments were repeated three times and the data were expressed as mean ± standard deviation (SD).

## Results

### Acute dermal irritation test

A skin irritation test of *C. asiatica* hydrogel formulation was performed on three rabbits. The calculation of Primary Dermal Irritation Index and the Grading of Irritancy Potential were conducted to indicate the acute dermal irritation of the tested samples. Based on the calculations, the asiaticoside rich hydrogel caused no irritation to the rabbit’s skin (the index was 0). Hence, the hydrogel formulation appeared to be biocompatible, safe and suitable to be used as a wound dressing.

### Incisional wound healing

**Fig. 2:**
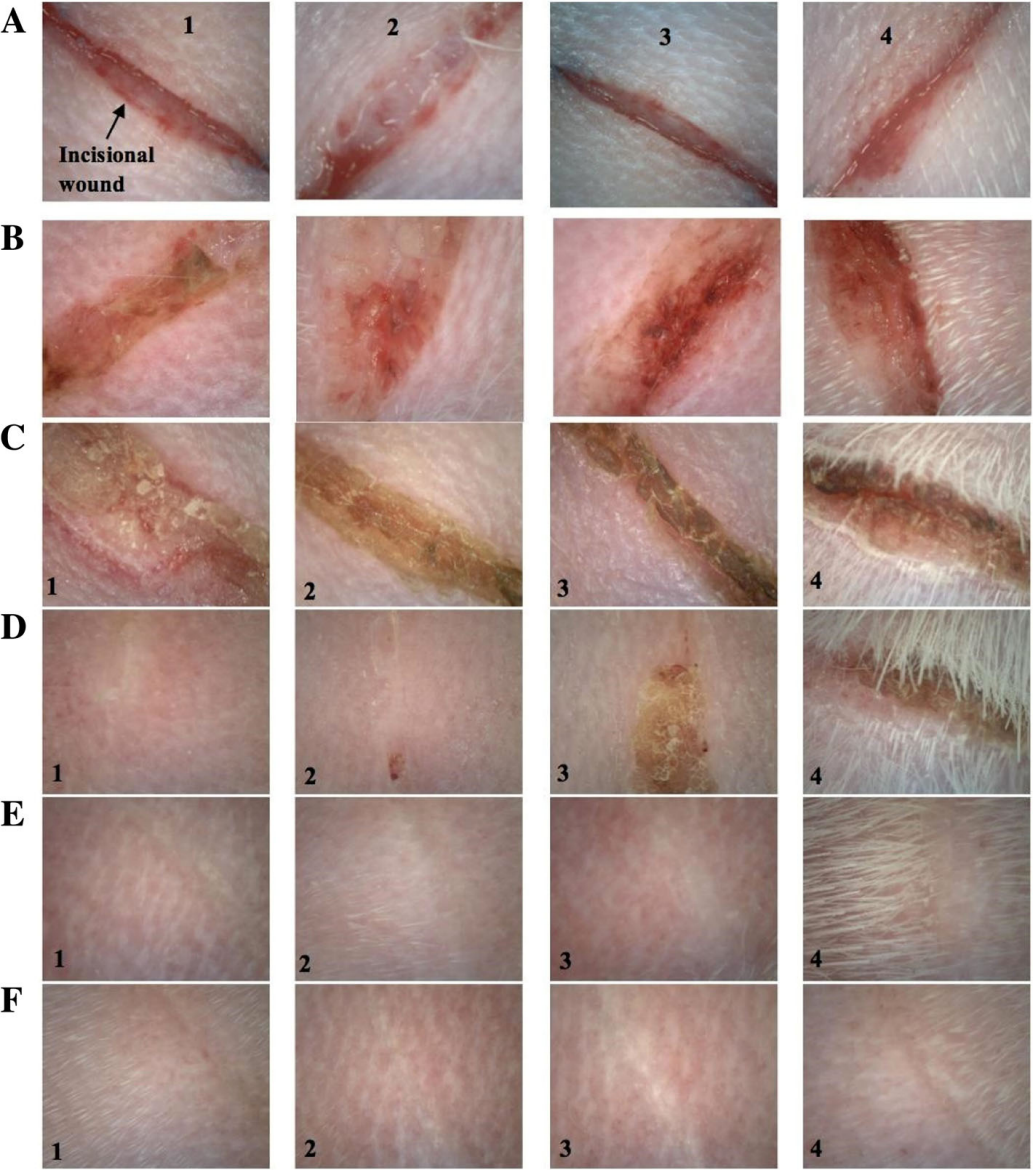
Photographs of each wound treated with (1) *C. asiatica* hydrogel, (2) Control (Madecassol cream), (3) Blank hydrogel and (4) Control (no treatment) at (**a**) 0 days (**b**) 1 days (C) 3 days (**d**) 6 days (**e**) 9 days and (**f**) 12 days post-operation

The wound healing efficacy of asiticoside rich hydrogel was estimated by applying the hydrogel to the created incisional wounds on the rabbits (Fig. 2). Qualitatively, all wounds showed a reduced wound size indicating that contraction subsided and epithelialisation took place as the experiment completed on Day 12. The results obtained on the Day 1 post treatment showed a little of inflammation and swelling around the wound area. At Day 3 post treatment, reduction in wound size were observed in all the animals on an average with the asiaticoside rich hydrogel showing much higher reduction in wound area as compared to the other groups. It was also observed that the swelling around the wound area was decreased as compared to the Day 0 and Day 1 in all the animals. As expected the wounds treated with drug loaded hydrogels showed no sign of dry scar formation instead the wounds were moist and this may be the only reason as to why the hydrogel treated wounds showed a better wound reduction as compared to the marketed formulation (Madecassol cream), blank hydrogel and the negative control (no treatment). At the end of 5th day post treatment the asiticoside rich hydrogel treated wounds showed a total wound closer and a formation of thin epidermis. This formation of epidermis was only found in the wounds treated with hydrogels. This can be attributed to the property of the hydrogel to maintain a moist environment in the wound region. At the end of Day 9 post treatment nearly all the wounds were healed and the epidermis layer showed thickening. At Day 12 post treatment, all the treated groups showed a similar contraction of the wound area wherein all wounds were observed to heal completely (Fig. 2).

### Period of epithelization

The period of epithelization indicates the time taken to form a complete epithelium over a denuded surface. It is expressed as the required days for the eschar (dead-tissue remnants) to fall without a remaining raw wound. If the epithelization period is fast, then the regeneration process will be fast. Likewise, if the epithelization period is slow, a scar will form over many weeks, even months. Table 1 represents the period of epithelization for all the groups. The fastest epithelization period was observed in asiaticoside-rich hydrogel group, followed by positive control group, blank hydrogel group and the control group. Statistically, it was found that there was a significant difference (*p* <  0.05) in the epithelization among asiaticoside-rich hydrogel and the other groups. Table 2 illustrates the *p-*values of the different treatment groups.

**Table 1:** Period of epithelization (Days)

#Rabbit (M = Male)	CAEMF Hydrogel	Control (Madecassol cream)	Blank Hydrogel	Control (no treatment)
M1	5	6	8	9
M2	6	6	8	9
M3	5	7	8	9
M4	6	7	9	11
M5	5	6	8	10
M6	5	7	9	10
Mean ± SD	5.33 ± 0.52	6.50 ± 0.55	8.33 ± 0.52	9.67 ± 0.82

**Table 2:** *p-*values of the treatment groups

Treatment groups correlation		*p* value
CAEMF Hydrogel	Positive control	0.021
	Blank hydrogel	< 0.001
	Negative control	< 0.001
Positive Control	Blank hydrogel	< 0.001
	Negative control	< 0.001
Blank Hydrogel	Negative control	0.007

## Discussion

Wound contraction increased rapidly on the following days for the respective group 3 and 4. Wound contraction of group 2 (positive control; Madecassol cream) and Group 1 (asiaticoside-rich hydrogel) was observed on day six. The reduction of wound treated with asiaticoside-rich hydrogel (Group 1) was approximately 40% higher compared to the untreated wound (Group 4). Data for the period of epithelisation may have some limitations since the measurement of wound size was recorded by day, and not by viewing the actual reduction event, and, therefore, may explain the wound condition better. Epithelisation period indicates complete formation of epithelium over a denuded surface, faster epithelisation periods help the regeneration process of healing and vice versa. Asiaticoside-rich hydrogel induced faster epithelisation period in comparison with the respective positive (Madecassol) and negative (vehicle) control groups.

*Centella asiatica* contains mixture of three principle ingredients namely asiaticoside, asiatic acid and madecassic acid has been formulated in hydrogel for the treatment of wound [31]. In that study, the titrated extract of *C. asiatica*-containing hydrogel was reported to heal wound on the Day 9 post treatment. In comparison, the present study used asiaticoside rich hydrogel that was obtained from the methanol extract of *C. asiatica* and the results demonstrated the ability of the hydrogel to exert complete wound healing in about five days (at the end of Day 5). Moreover, the type of hydrogel utilized by Hong et al. [31] (Na-deoxycholate (Na-DOC hydrogel) was different from the one used in the present study (polyvinyl alcohol/polyethylene glycol (PVA/PEG) hydrogel). The PVA/PEG hydrogel formulation and optimization has been used by other authors [9].

Asiaticoside wound healing activity might be associated with modulation of several mechanisms of action. Shukla et al. [32] have reported on the ability of asiaticoside to augment stimulation of antioxidant levels at an early stage of healing process which might be an important contributory factor in the healing properties of this substance. On the other hand, Kimura et al. [26] reported on the ability of asiaticoside to promote angiogenesis during skin wound repair by stimulating the vascular endothelial growth factor (VEGF) production as a result of the increase expression of monocyte chemoattractant protein-1 (MCP-1) and interleukin-1β (IL-1β) in the respective keratinocytes and macrophages induced communally by asiaticoside plus MCP-1. Lu et al. [33] reported on the ability of asiaticoside to promote fibroblast proliferation and extracellular matrix (ECM) synthesis, which are known to play important role in wound healing.

## Conclusion

Asiaticoside-rich hydrogel formulated in this study was found to be safe and biocompatible formulation with good physicochemical properties, which is suitable for the topical wound healing applications.

## Data Availability

The supporting materials can be obtained upon request via email to the corresponding author.

## Abbreviations

DCM: Dichloromethane
IL-1β: Interleukin-1β
MCP-1: monocyte chemoattractant protein-1
PEG 400: Polyethylene glycol 400
PVA: Poly(vinyl alcohol)
PVA/PEG: Polyvinyl alcohol/polyethylene glycol
VEGF: Vascular endothelial growth factor
VLC: Vacuum liquid chromatographic
